# Perceptions of human papillomavirus vaccination of adolescent schoolgirls in western Uganda and their implications for acceptability of HPV vaccination: a qualitative study

**DOI:** 10.1186/s13104-017-2749-8

**Published:** 2017-08-30

**Authors:** Andrew Kampikaho Turiho, Elialilia Sarikieli Okello, Wilson Winstons Muhwezi, Anne Ruhweza Katahoire

**Affiliations:** 1 Department of Psychiatry, School of Medicine, Makerere University, Kampala, Uganda; 2 Child Health and Development Center, Makerere University, Kampala, Uganda

**Keywords:** Perceptions about HPV vaccination, Adolescent schoolgirls, Acceptability, Uganda, Qualitative study

## Abstract

**Background:**

Human papillomavirus (HPV) vaccination has been perceived in diverse ways some of which encourage its uptake while others could potentially deter its acceptability. This study explored community member’s perceptions about HPV vaccination in Ibanda district and the implications of the perceptions for acceptability of HPV vaccination. The study was conducted following initial vaccination of adolescent schoolgirls in the district between 2008 and 2011.

**Methods:**

This qualitative study employed focus group discussions (FGDs) and key informant interviews (KIIs). FGDs were conducted with schoolgirls and parents/guardians and KIIs were conducted with school teachers, health workers and community leaders. Transcripts from the FGDs and KIIs were coded and analyzed thematically using ATLAS.ti (v. 6).

**Results:**

The HPV vaccination was understood to safely prevent cervical cancer, which was perceived to be a severe incurable disease. Vaccinations were perceived as protection against diseases like measles and polio that were known to kill children. These were major motivations for girls’ and parents’ acceptance of HPV vaccination. Parents’ increased awareness that HPV is sexually transmitted encouraged their support for vaccination of their adolescent daughters against HPV. There were reports however of some initial fears and misconceptions about HPV vaccination especially during its introduction. These initially discouraged some parents and girls but over the years with no major side effects reported, girls reported that they were willing to recommend the vaccination to others and parents also reported their willingness to get their daughters vaccinated without fear. Health workers and teachers interviewed however explained that, some concerns stilled lingered in the communities.

**Conclusions:**

The perceived benefits and safety of HPV vaccination enhanced girls’ and parents’ acceptability of HPV vaccination. The initial rumors, fears and concerns about HPV vaccination that reportedly discouraged some girls and parents, seemed to have waned with time giving way to more favourable perceptions regarding HPV vaccination although the study still found that a few concerns still lingered on and these have implications for HPV vaccination acceptability.

## Background

Despite the growth in literature on HPV vaccination, very few studies exploring changing perceptions of parents and other stakeholders following the introduction of HPV vaccination have been conducted in developing countries generally and in sub Saharan Africa in particular. Previous studies conducted in low resource settings [1] prior to the introduction of HPV vaccination found that understanding of cervical cancer and HPV was limited but sentiments toward cervical cancer vaccination were positive. There were concerns about quality of delivery; safety, adverse effects, and the effects of the vaccination on girls’ fertility.

The majority of studies that have explored community perceptions of HPV vaccination elsewhere have mainly used cross sectional quantitative surveys [2–8]. Very few qualitative studies have been conducted following the introduction of HPV vaccination. A qualitative study among Puerto Rican mothers and daughters after introduction of the HPV vaccination in the country reported inconsistent beliefs about susceptibility to HPV infection and cervical cancer; concerns about HPV vaccine effectiveness, safety, side effects and of the possibility that the HPV vaccination could promote sexual disinhibition [9]. Another qualitative study among a multiethnic sample of young women (13–27 years) in Malaysia reported that while participants were generally in favor of the HPV vaccination, concerns were raised regarding the vaccine’s safety, the potential for those who get vaccinated to be perceived as promiscuous and sexually active, and the vaccine being allowed by Islamic Law [10]. A qualitative study was conducted in Uganda prior to the introduction of the HPV vaccination [11] but none have been conducted since the actual introduction exploring how these perceptions have changed and their implications for long term acceptability of HPV vaccination. A similar study was done in neighbouring Tanzania [12].

Acceptability studies of the HPV vaccination indicate an overall positive response towards vaccination of young adolescent girls against human papillomavirus (HPV) [11, 13–15], a sexually transmitted virus that is implicated in the cause of cervical cancers [16, 17]. At the same time however, these studies also consistently highlight negative attitudes and concerns about vaccinations generally and HPV vaccination specifically [3, 6, 18]. The more common concerns are about safety, unknown side effects and whether the HPV vaccination actually protects against cervical cancer [2, 3, 5, 12, 19–22]. Among the worries reported in previous studies include the fear that HPV vaccination could result in an increase in early sexual activity and promiscuity among the vaccinated adolescents [3, 23, 24] and the perceived lack of adequate information about the vaccine [3, 5, 20]. These studies have mainly involved parents [3, 5–8]. Very few have involved adolescents or college-age women (18–26 years) and have been mainly quantitative in design [2, 20–22].

This study was conducted nearly 5 years after the initial introduction of the HPV vaccination in Ibanda district creating an opportunity to study how community perceptions about HPV vaccination may have changed over time. The study explored the community’s initial reactions to the vaccinations, how they changed over time and the implications of their perceptions for acceptability of HPV vaccination of adolescent girls in the district.

Between 2008 and 2011 adolescent girls in primary schools in Ibanda district were vaccinated annually against HPV. This was part of a demonstration project by the Uganda Government and Program for Appropriate Technology for Health (PATH) to evaluate different HPV vaccine delivery strategies for low-resource settings [25]. Mobilization and sensitization of communities prior to the introduction of the HPV vaccination and during the vaccinations took place at district, school and village levels. Posters and other IEC materials were distributed and radio messages aired. In addition, teachers and health workers were trained and given additional materials for educating others in the community as well as adolescent girls in schools about HPV vaccination. The IEC materials prepared for school children explained the HPV vaccinations in greater detail including how HPV is transmitted and why there was need for HPV vaccination of young adolescents as a protection against cervical cancer [26].

Regarding theoretical framework, this study was anchored in the first two components of the symbolic interactionism theory (Blumer, H., 1969 in Jeon, Y-H, 2014) [27]. The study also borrowed three of the five Health Belief Model (HBM) constructs namely; *perceived benefits, cues to action, and barriers,* which determine the likelihood that an individual engages in a given health enhancing behaviour [28]. The first component of the symbolic interactionism theory asserts that, ‘human beings act towards things on the basis of the meanings that the things have for them’. In this study, it was anticipated that communities acted towards HPV vaccination (accepted or declined it) on the basis of its meaning to them or their understanding of it. According to the second component, ‘the meaning of such things is derived from, or arises out of, the social interaction that one has with one’s fellows’. In this study, it was expected that community members’ understanding of HPV vaccination and their actions/attitudes towards it were shaped by their exposure to information about HPV vaccination during mobilization for HPV vaccination as well as their interaction with fellow community members. According to the HBM, *perceived benefits* refers the extent to which the individual believes that the various available actions are effective in reducing the threat, whereby if an action is regarded to be effective in preventing a disease, the likelihood of accepting the action is higher than if this is believed not to be the case; *cues to action* could be internal cues like bodily symptoms, or external cues such as the death of a friend, social influences, or exposure to a mass media campaign that may sometimes trigger appropriate health behaviour; and *perceived barriers* applies to the potential negative aspects of a particular health action that may function as impediments to undertaking the recommended behaviour. This study explored community perceptions of the HPV vaccination and their perceived implications for acceptability of HPV vaccination of adolescent girls in Ibanda district.

## Methods

### Study design and site

This qualitative study was carried out in Ibanda district between November and December 2011 using focus group discussions (FGDs) and key informant interviews (KIIs). Four FGDs were conducted with school girls and five with parents and guardians. In addition, 10 KIIs were conducted with teachers, health workers and community leaders. All key informants were involved in the mobilization and sensitization of communities about the HPV vaccination.

### Study participants

The girls, aged 13–16 years had received at least one dose of the HPV vaccination, were in primary five (P 5) or primary six (P 6), and were registered in their respective schools’ vaccination registers. The government-recommended age for starting primary school is 6 years. However, the policy on age for starting primary school is apparently not strictly followed. Some girls begin school late and girls’ education is often interrupted; hence the unexpected age-range of the study participants. At the time of data collection, the girls in P 6 had been vaccinated for 1 year and their P 5 colleagues had been vaccinated a month earlier. The parents had daughters that were fully or partially vaccinated against HPV. The teachers were designated ‘Senior Women’ in their respective schools and had been with the schools for at least 2 years. The health workers had been involved in reproductive health activities in health facilities that participated in HPV vaccination. Community leaders were sub-county-level local council (LC) III Secretaries for Social Services.

### Sampling

**Fig. 1:**
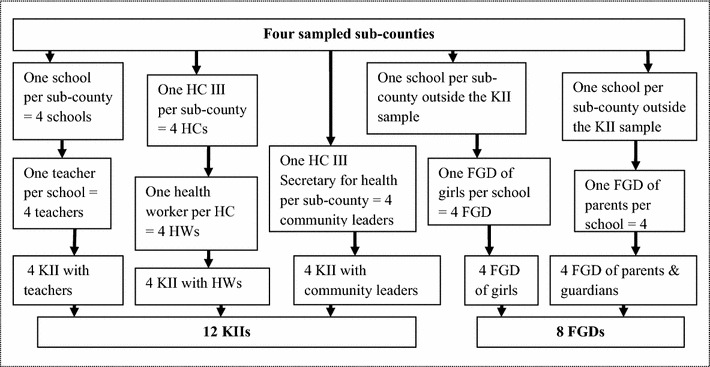
Sampling procedure. It is a diagrammatic presentation of the procedure that was followed to arrive at the sample of study participants

Study participants were purposively selected from four sub-counties out of 9. The projected population of Ibanda district in 2012 was 265,461 people (or average of 29,596 per sub-county) at an annual growth rate of 2.5% compared to the national average of 3.2%. In 2009, Ibanda had 272 primary schools (or approximately 31 primary schools per sub-county) of which 127 (47%) were government-aided. There were 54,094 children enrolled in primary schools (or approximately 6011 children per sub-county) 51.6% of whom were female compared to 49.4% male [29]. During sampling for this study, one school was selected from each sub-county and one teacher (Senior Woman) selected from each of the schools. Choice of health workers was influenced by choice of schools for key informant interviews. Once a teacher was recruited as a key informant in a school, one health worker was selected from the nearby health facility that was responsible for delivery of the HPV vaccine to that school. A community leader was also purposively selected in each of the four sub-counties. Key informants (KIs) of all categories and FGD participants were identified with the help of school teachers. Parents/guardians were recruited from areas near the schools sampled for girls’ FGDs in order to minimize inconvenience to the concerned individuals. In all, other than the schoolgirls, the groups were chosen because they were considered to be knowledgeable about adolescents and they were regarded as influential in the communities [30]. Saturation determined the sample size, as no new information would be generated from additional respondents. Twelve key informants were sampled but 10 were instead interviewed. These included 3 health workers, 2 community leaders, and 5 teachers. A total of 43 schoolgirls and 52 parents participated in FGDs. The sampling procedure is presented diagrammatically in Fig. 1.

### Data collection

The FGDs of schoolgirls and of parents/guardians were conducted separately using pre-tested and translated FGD guides. The number of participants in FGDs ranged from 8 to 12 per group. With the help of teachers, FGDs were held in classrooms and precautions were taken to minimize external disruptions. Each FGD was conducted by a moderator assisted by a note-taker. In addition to the written notes of the FGD, the proceedings were tape-recorded with the assent/consent of participants. Each FGD lasted approximately one and a half hours. Key informant interviews (KIIs) were conducted using a KI guide. The KIIs were conducted at sub-county offices, health centers and schools. Interview venues were carefully chosen in consultation with respondents to ensure privacy and confidentiality. On average, a key informant interview lasted for 45 min. The FGDs and KIIs explored the initial reactions of the community to the introduction of the HPV vaccination, community members’ understanding of the benefits of the HPV vaccination, fears, complaints and rumors about the HPV vaccination. They also explored girls’ willingness to be vaccinated, parents’ willingness to have their daughters vaccinated against HPV and the implications of these perceptions for HPV vaccination acceptability. Questions that were asked to the different categories of study participants and the respective data collection methods used are presented in Table 1.

**Table 1:** Questions asked to different categories of study participants and data collection methods used

Question	Study participant and data collection method		
	Schoolgirl (FGD)	Parent/guardian (FGD)	Health worker, teacher and community leader (KII)
What do you think the people understood to be the benefit of the HPV vaccination? *(E.g. protection against HPV/cervical cancer, HIV, other STIs)*	√	√	
How would you describe the initial reaction of the community to introduction of the HPV vaccine?			√
What fears about HPV vaccine have you heard about in the schools or community? *(I.e. fears among parents, fears among girls)*	√	√	√
What fears about vaccines in general have you heard in the schools or community?		√	√
How do you think the fears about the vaccine affected its acceptance?		√	√
What complaints did you hear of concerning the HPV vaccine? *(I.e. complaints by parents, complaints by girls)*	√	√	√
How do you think the complaints about the vaccine affected its acceptance?		√	√
What unfounded rumors or misinformation have you heard about the vaccine?			√
How do you think the rumors or misinformation influenced the behaviour and attitudes of adolescent girls about the vaccine?			√
How do you think the rumor or misinformation influenced the behaviour and attitudes of parents about the vaccine?			√
To what extent were girls willing to be vaccinated against HPV?	√		
What do you think made the girls willing to be vaccinated?	√		
What do you think made some girls not willing to be vaccinated?	√		
To what extent were parents willing that their daughters be vaccinated?	√	√	√
What do you think made the parents willing that their daughters be vaccinated?	√	√	√
What do you think made some parents not willing for their daughters to be vaccinated?	√	√	√
If there is vaccination against HPV in future, would you be willing for your own daughters to be vaccinated? *(Probe why YES and why NO)*	√	√	
Why would you be willing for your daughters to be vaccinated against HPV?		√	
Why would you be not willing for your daughters to be vaccinated against HPV?		√	
Would you advise your friend to get the vaccination if there is vaccination against HPV in future (*Probe why YES and why NO)*	√		
If there is vaccination against HPV in future, would you (and your fellow health workers, teachers, and community leaders) encourage and support young girls to be vaccinated?			√
Why would you encourage and support young girls to be vaccinated?			√
Why wouldn’t you encourage and support young girls to be vaccinated?			√
How do you think the perceptions about protection offered by the vaccine affects behaviour of the girls who receive the vaccine now and in future?		√	

### Data management and analysis

In order to minimize the risk of losing data, proceedings of each FGD were both hand-written and voice-recorded. Each KII on the other hand involved taking two sets of hand-written notes, one by the interviewer and the other by the note taker. These were later word processed by an assistant and merged. The voice recorded FGD data was transcribed and word processed. All word processed data was edited to ensure good quality. For each FGD, the first author read the word processed field notes and transcriptions and then merged the two to obtain a full record of the respective FGD. For each KII, the first author read the word processed notes of the interviewer and those of the note-taker before merging them to obtain a complete record. The first author developed a data analysis plan based on the objectives of the study. The first author carefully read the complete data for each KII and FGD and developed codes and themes using the constant comparison method. The data sets were coded and analyzed using the ATLAS.ti (v. 6). The codes were used to retrieve segments of the data. Memos were written describing the patterns and variations in the different segments of retrieved data. Verbatim quotations from the data were used to highlight key study findings. Additional verbatim quotations that are not cited in the article are presented in Table 2.

**Table 2:** Additional verbatim quotations concerning perceptions about HPV vaccination and their implications for cervical cancer prevention

Theme	Quotation	Source
*Perceptions related to efficacy of HPV vaccine*		
	Many children here used to die from the 6 killer diseases before immunizations began; but the children are now healthy… Around the year 1989, a family here lost four of its members to measles and we all saw it. Who do you think can stop their child from any vaccination after that incident?	FGD of parents at School 5
	We have learnt from experience that vaccinations work; especially against polio and measles. We no longer see many cases of those diseases in villages. So we are willing to bring our daughters in future. We want them to be healthy	FGD of parents at School 2
	We did not get a chance of being vaccinated, but now that the chance is here for our daughters, let us embrace it; after all we have seen many of our relatives and friends dying of cervical cancer… We all know of people who have died of cervical cancer in our villages	FGD of parents at School 1
	My mother died of cervical cancer; I wouldn’t want to see more deaths due to the same disease	FGD of parents at School 3
	Many deaths of women due to cervical cancer have occurred in the communities. This works as a live example to the community members that cervical cancer really kills. It has motivated parents to accept that their children be vaccinated to prevent such deaths in future	KII with a health worker at B HC II
	I was well taught by my teachers and the health workers who came to our school that the HPV vaccine would protect me from cervical cancer, which can lead to failure to produce children… With HPV vaccination, we shall have healthy lives in future and be able to give birth to children… Parents encouraged us to be strong and get the injection in order for us to be healthy in future	FGD of girls at School 4
	We understood HPV vaccination to be for preventing cervical cancer so that we can have healthy reproductive life… Our parents accepted because they wanted us to have healthy life, free of cervical cancer	FGD of girls at School 5
	Our girls are lucky to have this chance… They have peace of mind because they are confident that their future is free of the cervical cancer disease… We are happy about the vaccination because it saves our money that would be spent treating cervical cancer in future	FGD of parents at School 2
	Some women fail to get screened (for cervical cancer) because of long queues (at Ibanda Hospital). For instance I have been to Ibanda Hospital three consecutive times seeking cervical cancer screening services without success due to the large numbers of women who go there for screening	KII, Senior Woman at School 1
	Large numbers of mothers are turning up at Ibanda Hospital for cervical cancer screening… Other than the increased demand for cervical cancer screening services, many mothers are also inquisitive whenever they visit health centers; they are eager to know when their children are due to be vaccinated so that they don’t miss the HPV vaccine…	KII, Health worker at B HC II
	We shall allow our future children to go for HPV vaccination because we would want them to live healthy lives like us who have been vaccinated… We would advise our friends to go for HPV vaccination because it is a good way of ensuring a healthy future of a woman	FGD of girls at School 2
	Of course we would encourage the girls to be vaccinated since cancer has no cure	KII, Senior Woman at School 6
	We are so much aware that the vaccination cannot prevent other STDs. We know that HPV can be transmitted through sex and that use of condoms during sex may not fully prevent the virus	FGD of parents at School 2
	The nurses told us to avoid sex because that is how the disease is spread from men. They also advised us to avoid sharing knickers with our friends because the disease could also be spread that way	FGD of girls at School 2
	The risks to young girls who have sexual intercourse (with men or boys) are largely the same for all girls… They may get HIV and other sexually transmitted diseases such as gonorrhoea, and now HPV… They usually get pregnant	FGD of parents at School 4
	Since these girls were sensitized and educated about the different ways of transmission of HPV, some have changed their behaviour… They are showing less sexual activity because they fear contracting HPV	FGD of parents at School 3
	Some girls think that HPV vaccination can protect them from getting pregnant and that is why they go around having sex with men… The girls say that since they received the HPV vaccine, they cannot get pregnant when they sleep with men	FGD of girls at School 4
	Other parents believed that this vaccine would reduce the severity of cervical cancer in case one goes ahead to suffer from it even after the vaccination… That cervical cancer would not weaken the girls who have taken the HPV vaccine…	FGD of parents at School 3
	No fears have been recently reported about vaccines. In fact, people have been actively involved in immunizing their children against diseases like polio, measles, and hepatitis	KII, Senior Woman at School 1
	Some girls complained that their arms got swollen because of the HPV injections but this did not take long. We thought it was just like other injections where the pain would later disappear	FGD of parents at School 4
	The girls told us on the day of vaccination that they were feeling some pain in the arms that had been injected but a few days later, they told us that they were fine	FGD of parents at School 2
	I first feared taking the injection because I thought my arm would swell and I end up not doing any work or going to school… I thought I would bleed very much after the injection but there was no bleeding… At the first injection, I was worried but for the second one, I was confident and not fearing anything	FGD of girls at School 5
	I am not aware of any fears in the community about vaccines in general; no complaints and no rumours about HPV vaccine…	KII with Senior Woman at School 9
	Other than the initial rumor of possible sterilization effect of the HPV vaccine, no fears, rumors or serious complaints about HPV have so far been reported both at school and in the community…	KII with Senior Woman at School 8
	Some parents still hold old beliefs that vaccinations make children sick. At first, they did not allow their children to get the HPV vaccine; but when the HPV vaccine was explained to them they understood and cooperated	FGD of parents at School 4
	People at first feared the HPV vaccination… In early 1990s children were immunized against Polio in Ibanda. About 3 months later, children started dying of fever and high temperatures. Parents here thought that maybe the vaccine was deliberately administered to their children to kill them. Consequently when the HPV vaccine was introduced, parents were suspicious that the vaccine could be meant to harm their children. Besides that concern, there was no other negative perception of the HPV vaccine	KII, Senior Woman at School 6
	Nothing is going to stop the vaccination. But maybe with time it will depend on the health of those who have been vaccinated. About 10 years ago, many children died here shortly after being vaccinated against Polio. Only such a scenario would stop the HPV vaccination if it happened among the HPV vaccinated girls	KII, Senior Woman at School 9
	They said it kills a woman’s eggs and she does not produce children… Some people got scared after being told in the villages that people who get vaccinated against HPV will not produce children in future… We heard that those who are vaccinated will in future die while delivering children and that worried us… Many of us were worried; they had many disturbing questions in their minds… Some believed that the vaccine was meant to reduce fertility of women in future by destroying their ovaries… That it might be aimed at preventing birth to many kids; a means of reducing the population…These were the rumours spreading in the villages…	FGD of parents at School 4
	We heard that the injection kills our eggs so that we don’t produce children in future;…I got worried because I was told I would produce only twins after taking that injection… With the HPV vaccination, we can have safe births in future. The vaccine strengthens the uterus and keeps it safe for normal pregnancy and child birth	FGD of girls at School 2
	Some people wondered why it was young girls of a specific age being vaccinated and not mature women… They thought the vaccination was a trick by government to prevent over-population by injecting girls with medicine that would prevent them from giving birth in future… There was rumor that the vaccine damages a girl’s ovaries so she can’t produce children in future. But it was later explained and understood that the vaccinations were meant to prevent cervical cancer	FGD of parents at School 5
	One woman came and asked me if it was true that the vaccine meant to sterilize their daughters… I educated her about the vaccine and she went back very happy and willing to encourage the young girls to go for HPV vaccination	KII, Health Worker at D HC III
	When the HPV vaccine was first introduced, many people here in Kabagoma had a belief that the vaccine was meant to sterilize women… But this perception changed when a woman in this very village died of suspected cervical cancer. During the funeral service, the health worker who used to treat the deceased used the opportunity to explain to the mourners the suffering and death from cervical cancer… From then onwards, all people got converted…they had no more misgivings about the HPV vaccine	KII, Senior Woman at School 1
	Two girls missed the vaccination because their parents discouraged them… Their parents told them that if they got the injection they would never produce children in future… Some parents were not willing that their daughters be vaccinated because of the belief that the vaccination causes heavy bleeding during the menstruation… That the vaccine can cause pain in the uterus	FGD of girls at School 5
	Parents in Kicuzi Sub-county barred their children from getting the vaccine to the extent of refusing them to go to school until the RDC and CAO intervened… Those parents had heard rumours that the HPV vaccine would sterilize their children or kill them…	KII, Health Worker at B HC II
	The effect of fear of pain of injection was witnessed here in this school where three girls from the same family missed to be vaccinated… I followed them up and even threatened their mothers that they would be arrested if they didn’t take the girls to be vaccinated. Since the parents saw no serious problem with the vaccine they later took the girls to the health centre and they got the vaccine	Senior Woman at School 1
	Vaccines are poisons to make people get the diseases against which they are vaccinated and ultimately die… We heard that the vaccine would in future cause cervical cancer to those who receive it…. but when the health workers came and explained it well it was understood	FGD of parents at School 4
	They said the vaccine would in future cause disease to those who receive it… That the injection contains chemicals that kill a person gradually; but the health workers came and changed this	FGD of girls at School 4
	Although many parents were willing to have their daughters vaccinated, few others were not willing (initially) for fear of losing their daughters; they believed that the vaccination could cause death at some point in future	FGD of schoolgirls at School 5
	It was said that when the HPV vaccine is given to children, it weakens their intellect so that they do not study beyond primary seven… They remain with capacity to vote for politicians but cannot question whatever the politicians say	FGD of parents at School 3
	I think we are not in position to comment about long term effects of the HPV vaccination now because the vaccination has just been done yet the effects could be seen many years in future… We see the girls are living normal lives; may be in future we would have observed something strange and we report it	FGD of parents at School 2
	Some religions such as… advise their believers not to take their children for vaccination… Cult members believe that their God protects the children; so they do not see need to vaccinate their children	FGD of parents at School 3
	Sometime in the past,… of the Great African Radio in Mbarara led a campaign against vaccination against the ‘Six killer diseases’. That campaign later contributed to the initial reluctance of some parents to allow their children to get the HPV vaccine. But the parents later accepted after being sensitized about the HPV vaccination	KII, Senior Woman at School 5
*Questions and uncertainties about HPV vaccine*		
	Vaccination was done only in schools. Why not even in the villages so that those girls who are not in school can also access it? That is what bothers me	FGD of parents at School 4
	When the fist vaccination took place in this school, I was studying in Jason Primary School, a Pentecostal private school where the vaccination did not take place. When I later changed to this school …the health workers came to deliver the second vaccine and I took it and the next one. I don’t know whether there is a problem with that… At the time the first injection was given, I was in hospital looking after my sick mother. So I managed to take the last 2 doses but missed the first one. When will I get what I missed?	FGD of girls at School 3
	All girls should be vaccinated; both those who are below the age of 9 and those who are above the age of 9. Even girls in lower classes of P3, P2, and P1 should be vaccinated because some girls start school when they are old	KII, Senior Woman at School 9
	Since cancer does not have a cure and there is now a way of preventing it, all girls should be vaccinated… Girls of all ages should be vaccinated to protect them from this killer disease	KII, Senior Woman at School 8
	The HPV vaccination program should continue but should use the criterion of age and not class since there are some girls of eligible age who are in lower classes. Screening for the HPV vaccination should start from P 1 and not in P 4 or P 5 as is currently the case	KII, Health worker at D HC III

## Results

Findings from the study are presented using the three HBM constructs—*perceived benefits, cues to action and barriers*. The findings show that community members perceived HPV vaccination favorably in terms of benefits and cues to action although there were some misconceptions and concerns about vaccination of young girls against HPV that could potentially discourage parents and adolescent girls from HPV vaccination.

### Perceived benefits of vaccination of adolescent girls against HPV

Based on the messages used during the mobilization of the communities for the HPV vaccination, the HPV vaccination was understood to prevent cervical cancer which in itself was perceived to be a severe disease that otherwise had no cure. Parents, schoolgirls, teachers and health workers all reported that the HPV vaccination program was largely embraced in the communities because it was understood to prevent cervical cancer and safeguard the girls’ future health in general and reproductive health specifically. While some parents and schoolgirls thought the vaccination prevented cancer of the cervix, others especially girls linked the vaccination to their future reproductive health emphasizing that they would be able to give birth to children.

*“I was well taught by my teachers and the health workers who came to our school that the HPV vaccination would protect me from cervical cancer, which can lead to failure to produce children… With HPV vaccination, we shall have healthy lives in future and be able to give birth to children…” (FGD of schoolgirls)*

Health workers and community leaders explained that illness and deaths associated with cervical cancer witnessed in the communities were an eye-opener to parents that cervical cancer is fatal and its prevention is critical. Parents in the FGDs mentioned parents, relatives, friends, and other acquaintances that had suffered and died from what they perceived to be cervical cancer.

*“We have seen what happened to some of our friends who got cervical cancer… No parent would want to see their daughter suffer from that dangerous disease especially after learning about its prevention” (FGD of parents)*.

Compared to other study participants, however, schoolgirls barely expressed concern about the pain and death commonly associated with cervical cancer, but they were concerned that it jeopardizes a woman’s ability to bear children. They reasoned that by protecting girls against cervical cancer, the vaccine would be safeguarding the girls’ future reproductive health.

Community leaders as well as school teachers pledged to continue mobilizing parents and eligible girls to embrace HPV vaccination.

*“The vaccination program will be supported to the end… It will still be welcomed in future since it is known to help prevent cervical cancer that is a killer disease” (KII with a community leader).*

When they were asked if they would take their future daughters for the vaccination and encourage their friends to do the same, virtually all schoolgirls who participated in the FGDs responded affirmatively. This was a likely consequence of girls’ perception of the HPV vaccination as safeguarding the girls’ future health in general and reproductive health specifically by preventing cervical cancer.

*“We would let our daughters to get vaccinated because we want them to be healthy in future… and have children….” (FGD of schoolgirls)*

It was acknowledged in the FGDs with parents that the HPV vaccination prevents an otherwise costly, painful and deadly disease. Parents reasoned that the vaccinated girls would rest assured of a healthy life in future, free from cervical cancer. Parents also explained that the vaccination was likely to save resources that would otherwise be expended on caring for patients who might in future suffer from cervical cancer if they were not vaccinated. These perceptions positively influenced their attitudes towards the vaccination.

*“Our girls are lucky to have this chance… They have peace of mind because they are confident that their future is free of the cervical cancer disease… We are happy about the vaccination because it saves our money that would be spent treating cervical cancer in future” (FGD of parents)*

Most parents and schoolgirls who participated in FGDs understood that HPV vaccination prevents a sexually transmitted infection. This they explained encouraged parents to accept their daughters’ vaccination since parents expected their daughters to get married in future and they would be at great risk of contracting the virus in case they got married to men carrying the virus.

Other than the parents’ and girls’ positive perceptions of HPV vaccination of young girls, the study findings show some misconceptions of HPV vaccination that paradoxically were likely to encourage parents’ and girls’ acceptability of vaccination of young girls against HPV. Some girls believed that other than prevention of HPV infection, the HPV vaccination prevents; human immune deficiency virus (HIV) infection, Hemagglutinin Neuraminidase (HN) influenza virus infection, and pregnancy. An FGD of parents affirmed that some parents also believed that the HPV vaccination also prevents HIV infection. The source of that misinformation however could not be named although there were some suggestions from the parents as to the possible source of confusion.

*“Since HPV and HIV are both viruses, some people believed that HPV vaccination can also prevent HIV” (FGD of parents).*

Parents also indicated that other parents welcomed the vaccination program out of the mistaken belief that the vaccination would promote safe child births in future by strengthening the vaccinated woman’s uterus (not cervix). Others falsely believed that the vaccination reduces the severity of cervical cancer in case a vaccinated girl developed the disease.

### Perceived cues to action on HPV vaccination

Parents and girls largely perceived HPV vaccination favorably and embraced it based on their previous vaccination experiences. Prior perception that vaccinations in general effectively prevented diseases that were known to kill children motivated girls to be vaccinated against HPV and their parents to support them. Notably, parents pointed out that prior to the introduction of vaccination against polio and measles, many childhood deaths and disabilities occurred in their communities but the situation had almost been reversed by vaccination. They expected the HPV vaccination to bring about the same positive changes in relation to cervical cancer.

*“Many children here used to die from the 6 killer diseases before immunizations began; but the children are now healthy… Around the year 1989, a family here lost four of its members to measles and we all saw it. Who do you think can stop their child from any vaccination after that incident?” (FGD of parents)*

This was echoed by a community leader who observed that the success of the routine Child Days Plus (CDP) immunization of children against the ‘six killer diseases’ in Uganda had encouraged parents to accept the HPV vaccination.

Parents who had positive experiences with other childhood vaccinations considered HPV vaccination to be as safe as any other vaccination they were familiar with. The main complaints by vaccinated girls were pain and minor swellings around the injection points, which were reportedly characteristic of all injections and were familiar complaints to the parents.

*“Some girls complained that their arms got swollen because of the HPV injections but it did not stay long. We thought it was just like other injections where the pain would later disappear” (FGD of parents)*

Nearly all those interviewed in the study indicated that no major complaints or serious side effects had so far been reported about the HPV vaccinations despite the fact that in nearly all FGDs with parents and girls at least one negative experience was reported. These included; pain during injection, pain in the vaccinated arms after injection, swollen arms, altered menstrual cycles whereby menstruation lasted longer than usual, and persistent abdominal cramps. In one of the girls’ FGDs, a girl was cited to have got cramps that lasted for a month after the HPV vaccination: “*The cramps started in the lower abdominal region and spread to her right leg. The case was referred to doctors”.* In all however, the study participants reported that the negative experiences were mild and short term. According to the girls in FGDs, the experiences of girls who received the initial vaccinations showed them that the vaccination had no serious side effects and encouraged them to follow their example. As a result of girls’ understanding of HPV vaccination as having no serious side effects, those who participated in the study were generally willing to let their future daughters be vaccinated.

*“I first feared taking the injection because I thought my arm would swell and I end up not doing any work or going to school… I thought I would bleed very much after the injection but there was no bleeding… At the first injection, I was worried but for the second one, I was confident and not fearing anything” (FGD of girls)*

Most parents believed that HPV vaccination was safe and allowed their daughters’ to be vaccinated as a result.

*“Most of us encouraged our daughters to be vaccinated… We would be willing that our girls get vaccinated in future because no one has seen any adverse side effects of the vaccine” (FGD of parents)*.

Some parents initially feared that the vaccination would harm their children and refused to send them to school for the first dose of vaccination. But after the parents realized that the children who received the first dose were safe, they allowed their children to get the subsequent doses.

*“Some parents refused to send their children to school on the first day of vaccination and they missed the first dose. After realizing that the children who received the first dose had not died, the previously reluctant parents allowed their children to get the second and third dozes of the vaccine…” (FGD of parents)*

Key informants especially health workers and school teachers indicated that perceived safety of the HPV vaccine was a major motivation for parents’ willingness to have their daughters vaccinated against HPV.

### Potential barriers to HPV vaccination

Findings from this study revealed various continuing misconceptions about HPV vaccination. While some were reported to have been prior to the introduction of the HPV vaccination, it was evident that some had persisted even after several years of HPV vaccination and sensitization in the district. Most of the misconceptions and the concerns expressed about HPV vaccination were potential barriers to its sustained acceptability. They were likely to foment HPV vaccine hesitancy (delay in acceptance or refusal of vaccination despite availability of vaccination services) whose determinants have been grouped into three—contextual, individual/group, and vaccine/vaccination-specific influences [31]. Findings from this study fall under two categories—individual/group and contextual influences.

#### Individual and group influences

Information was disseminated during HPV vaccination sensitization in Ibanda showing that the vaccination prevented HPV infection. There remained however some uncertainty regarding effectiveness of the vaccination in preventing HPV infection and cervical cancer. Some of the parents were unsure of the vaccination’s long-term protection against HPV. They remained worried that their daughters might contract the HPV even after the vaccination. They argued that it was too early to tell if the vaccination was effective in offering protection against cervical cancer since the vaccinated girls were still young.

*“Cervical cancer occurs in mature women. We cannot know the effect of the HPV vaccine now when the girls are still young… We shall be able to know what we are dealing with in future when the girls start producing children” (FGD of parents).*

Both parents and girls indicated that people in the community were wondering if it was useful to vaccinate girls who had already initiated sex. Some parents suspected their daughters to have already started having sex by the time of vaccination. They thought that their daughters had most likely already contracted the HPV virus and they saw no point in the girls being vaccinated. They were not sure if one could remain HPV free even after initiating sexual intercourse. According to the girls, some parents reasoned that *“the vaccination will not help such girls anyway”*. Similarly, sexually active girls believed they already had acquired the HPV and did not go for vaccination against HPV.

*“I know one girl who believed she already had the HPV. She saw no reason to take the HPV injection” (FGD of schoolgirls).*

Misconceptions about safety of the HPV vaccination persisted despite dissemination of information about safety of HPV vaccination during sensitization. The fear that the HPV vaccination could have unspecified long-term adverse effects on the vaccinated girls featured in all FGDs of parents as well as in those involving girls. It was also reported in a KII with a school teacher. Some parents were said to have been reluctant to let their daughters get vaccinated due to those fears.

*“I think we are not in position to comment about long term effects of the HPV vaccination now because the vaccination has just been done yet the effects could be seen many years in future… We see the girls are living normal lives; may be in future we would have observed something strange and we report it” (FGD of parents).*

Schoolgirls reported that some of the vaccinated girls feared that the injection might cause long term physical damage to the vaccinated arms since some girls got swollen arms after the injection. They were worried that they would get paralyzed in the vaccinated arms due to the pain they felt during and immediately after injection. Others were worried that the injection point would get swollen and result into cracked skin. The fear was corroborated during some key informant interviews.

The rumor that HPV vaccination could in future jeopardize vaccinated girls’ reproductive health was recurrent across all categories of study participants—schoolgirls, parents, community leaders, school teachers and health workers. It was reported that although most of the girls were not worried about receiving the HPV vaccination, a few were concerned about the rumours that the HPV vaccination could cause infertility in girls.

*“People say that the HPV vaccine may make us fail to bear children in future; but we have also been told that it is not true that the injection can cause infertility. So we do not know the truth” (FGD of schoolgirls).*

One girl reported hearing that the vaccine had been deliberately made to cause death of the recipients or stop them from bearing children in future. Some parents were worried that the vaccination was intended to compromise future reproduction ability of the vaccinated girls by increasing other uterine infections. The rumor was that the HPV vaccination would cause the uterus to be hostile to HPV but compromise the immune system in the uterus, making it prone to other infections. Other parents were initially suspicious when they got to know that the vaccination was targeting young girls and excluding mature women. They thought it was a disguised population control measure by government whereby the vaccination would damage the girls’ ovaries so that they do not bear children in future.

*“Some people wondered why it was young girls of a specific age being vaccinated and not mature women… They thought the vaccination was a trick by government to prevent over*-*population by injecting girls with medicine that would prevent them from giving birth in future… But it was later explained and understood” (FGD of parents)*

Key informants affirmed that some parents initially perceived the vaccination as a measure to reduce their daughters’ future childbearing abilities; to sterilize or even kill them. Others reportedly thought that the vaccination was meant to reduce numbers of particular population groups.

Participants in FGDs of girls reported having heard that the vaccination would cause child birth complications and possible death during child birth. They reported a rumor that HPV vaccination prevented conception and child birth, and they were worried about potential menstrual effects like heavy bleeding and pain during menstruation. They also reported that they had heard that the vaccination would in future cause conception of twins in unexplained ways. Girls further reported a fear that HPV vaccination could be dangerous if administered during pregnancy. Participants in one girls’ FGD cited a pregnant girl whose parents stopped her from getting vaccinated against HPV because they feared for the safety of the mother and the unborn baby.

Across all categories of study participants, the fear that HPV vaccination could have adverse effects on reproductive health of the vaccinated girls was said to have impeded acceptance of HPV vaccination among both schoolgirls and parents. Some parents and schoolgirls who were interviewed in the study continued worrying that the vaccination could be dangerous to the girls’ future fertility despite the pre-vaccination sensitization that emphasized the contrary. All the key informants pointed out however that these misconceptions were largely corrected through sensitization.

Parents and health workers reported a belief in the communities that vaccines in general can sometimes have paradoxical effects. A rumor initially circulated in the communities that HPV vaccination could cause cervical cancer. However, both parents and health workers pointed out that the perception had largely changed after sensitization.

Parents cited a worry among parents that vaccines in general and HPV vaccine particularly, when administered to children suffering from diseases such as malaria, may worsen their conditions and even cause death. They also wondered if it would be safe to vaccinate girls who are HIV positive. They reported that some of their fellow parents as well as schoolgirls were initially worried by rumours that HPV vaccination would cause death in unexplained ways. They revealed a belief that vaccines are responsible for advent of previously unknown diseases locally. Parents also reported a rumor that the HPV vaccination was still experimental. It was believed that, the girls in Ibanda and Nakasongola Districts (HPV vaccine demonstration districts in Uganda) were being used as experimental guinea pigs to suffer in case of adverse side effects. However, this belief was said to have been neutralized by sensitization.

*“They said HPV vaccination would in future cause disease to those who receive it… That, the injection contains chemicals that kill a person gradually… That, some diseases were not common in the olden days; but after introduction of vaccines, people are suffering from all sorts of diseases; but the health workers came and changed this thinking…” (FGD of parents)*

Schoolgirls and parents disclosed a belief by parents that vaccines in general can compromise an individual’s mental power. Parents believed that the HPV vaccination was meant to lower their children’s intelligence so that their property could be grabbed in future by unspecified people. Parents also indicated that the vaccination exercise was somehow politicized; there was rumored connivance of local politicians with scientists to inject their children with a vaccine that would retard their intellectual development and render them politically subservient.

*“It was said that when the children get vaccinated against HPV, it weakens their intellect so that they do not study beyond primary seven… They remain with capacity to vote for politicians but cannot question whatever the politicians say” (FGD of parents)*

#### Contextual influences

Misconceptions about safety of the HPV vaccination were largely based on previous experiences of adverse effects of other vaccinations. Some parents were reported to believe that vaccinations in general cause illness of children and they initially feared that the HPV vaccination would be dangerous. FGDs with parents and key informant interviews with community leaders and school teachers indicated that initially there were some fears triggered off by previous negative experiences with vaccinations in Ibanda District. It was reported that in the early 1990s, many children died in the villages following a polio vaccination program. After being vaccinated, children who had previously been well would develop high temperatures, fever and other illnesses sometimes culminating into death. It is suspected that an expired vaccine was used. Some sections of the population believed that a defective vaccine was deliberately used to kill the children for unexplained reasons as explained by one of the parents in an FGD.

*“Vaccination was done (in Ibanda) some years ago and some kids died. We lost confidence in vaccinations after that incident… Some people still believe that vaccinations can kill…” (FGD of parents)*.

Nevertheless, the sensitization prior to HPV vaccination is said to have greatly improved the situation.

Parents and teachers indicated that some groups in Ibanda considered vaccinations in general as religious and cultural transgressions. Parents implicated two cult-like groups (names withheld) in that region of the country for notoriously discouraging their members to vaccinate their children. They indicated that the believers often don’t respect government vaccination programs; hence children of those believers may have missed the HPV vaccination. Parents and teachers also blamed a locally prominent radical traditionalist and Pan Africanist (name withheld) for having campaigned against all vaccinations via his FM radio. That campaign was said to have later contributed to the initial reluctance of some parents to allow their children to get vaccinated against HPV. Sections of the population were said to continue to harbor suspicions about vaccinations as a result of his influence although the sensitization prior to HPV vaccination was said to have largely neutralized his and the religious leaders’ influence.

## Discussion

Findings from this study showed that HPV vaccination was widely understood to prevent cervical cancer (and not necessarily HPV) and this perception contributed to acceptability of HPV vaccination. This finding has previously been reported by other studies [3–5, 21, 22] and adds credence to the argument and strategy of marketing the vaccine as a cervical cancer vaccine rather than as an HPV vaccine [32].

Community members’ attitudes about vaccinations in general were largely favourable based on observed ability of vaccinations to prevent their targeted diseases, which contributed to acceptability of HPV vaccination. This study also found that community members generally supported HPV vaccination based on the perception that it prevents a severe disease. The contribution of positive attitudes about vaccinations in general and of perceived severity of cervical cancer towards HPV vaccine acceptability have been previously reported [3, 6, 18, 32]. These findings suggest that effective promotion of vaccination against HPV ought to make clear reference to the success profile of other vaccinations. They also underscore the need to provide as much information as possible about the nature of cervical cancer targeting especially the adolescents who appeared to be less concerned about severity of cervical cancer in our study so as to enhance acceptability of HPV vaccination. In this study, vaccination against HPV was understood to carry long term economic benefits for the vaccinated individuals, their families and society at large. High cost of cancer care has been cited among the barriers to accessing cervical cancer care in Uganda and other developing countries [33, 34]. Some participants in this study also contended that HPV vaccination insulates the vaccinated individuals against the negative thoughts about possibility of developing cervical cancer. These perceptions about HPV vaccination are consistent with the observation that an effective vaccine against cervical cancer would reduce healthcare costs associated with cervical cancer and the negative psychological consequences of HPV-related diagnoses [35]. These findings suggest that marketing of the HPV vaccine ought to clearly articulate the economic and psychological costs of cervical cancer disease.

Paradoxically, in this study, awareness that HPV vaccination prevents a sexually transmitted infection encouraged support for HPV vaccination among parents. These findings contradict numerous studies which report fear among HPV-vaccination critics that the perception of HPV vaccination as vaccination against an STI could deter its acceptance out of concern that it could lead to adolescent girls’ early sexual activity [24, 30, 36, 37]. The findings support the argument that vaccination against HPV should be used as a key opportunity for increasing young people’s awareness of their risk to acquiring STIs when they become sexually active, and the need for prevention [14, 38]. Basing on these findings, future vaccination programs should clearly articulate the sexual transmissibility of HPV and the preventive role of HPV vaccination. This would likely further enhance HPV vaccination acceptability.

Consistent with documented evidence showing that the vaccines against HPV are safe [38–40], results of this study basically indicate that the vaccination against HPV was largely understood to have no serious adverse effects. The main complaints about HPV vaccination like pain and swelling were familiar to parents based on experiences with other vaccinations. These and other adverse effects like; dizziness and headache have been reported in other studies [41]. Moreover, these complaints are usually brief and non-serious [42]. The perception that the HPV vaccination is harmless was reported to have been a major incentive for its acceptance, which is consistent with results of other studies [5, 9, 37]. These findings suggest that efforts to maximize uptake of HPV vaccination should emphasize the safety profile of vaccines in general. “There is nothing inherent in either the bivalent or quadrivalent vaccine to suggest any future safety problems and there is no reason to expect long-term safety to differ from the well-documented safety profile of HBV vaccine” [38].

In this study, although vaccination against HPV was widely perceived as prevention against cervical cancer, some parents were uncertain about the long-term protection by the vaccination. This concern has been cited in other studies [9] yet all available evidence shows that the HPV vaccinations do prevent cervical cancer [4, 38, 39]. The concern underscores the need for future vaccination programs to incorporate messages emphasizing the ability of HPV vaccination to provide long-term protection based on proven long-term effectiveness of other vaccines. Some girls as well as parents in this study also questioned the usefulness of vaccinating girls who are already sexually active and pointed out that some eligible girls missed vaccination for that reason. This issue has featured in other studies [9]. HPV vaccinations are indeed not therapeutic; thus adolescents already infected with the targeted HPV types cannot receive protection [43]. Nonetheless, “the Center for Disease Control and Prevention’s Advisory Committee on Immunization Practices (ACIP) also recommends vaccination for sexually active females, regardless of a history of HPV infection or an abnormal Pap test because there appear to be no adverse effects from vaccinating women with prior HPV infection and because women infected with one type are still at risk for infection with others” [35]. This should be explicated during promotion of the HPV vaccination. Sections of the community also variously misunderstood the protection provided by HPV vaccination. Some perceived the vaccination to offer other protections including prevention against pregnancy and uterine cancer as well as protection from other virus infections such as HIV and the influenza virus—Hemagglutinin Neuraminidase (HN). HPV has often been mistaken with other sexually transmitted viruses such as the HIV and herpes simplex virus [44]. In this study, some of the misconceptions were said to have inadvertently enhanced acceptability of the HPV vaccination. Nonetheless, the misconceptions point to the need to clarify the protective limits of the vaccination because people have a right to be availed with correct information before consenting to undergo medical procedures.

This study further captured several misconceptions and concerns regarding safety of HPV vaccination. Some concerns such as; adverse effects of previous vaccinations, fear of unknown side effects, pain and swelling experienced during and after vaccination, and fear of possible long term physical harm to the vaccinated arms are realistic concerns but seem to have been exaggerated and misinterpreted by sections of the public to discourage uptake of the vaccine. However, there were also several misconceptions fuelled by outright rumor and misinformation for unclear motives. These include misconceptions that vaccination against HPV; causes reproductive health problems (including life-threatening childbirth complications, alteration of menstrual cycle, birth of twins, and infertility), can cause cervical cancer, can worsen pre-existent illnesses, can cause death and alien diseases, is still experimental, and can cause mental retardation. These misconceptions suggest lack of information about HPV vaccination and they tended to foment negative attitudes about it. This finding supports results of other studies showing lack of information as a factor for low acceptability of HPV vaccination [3–5, 20]. They point to a need for HPV vaccine promotional activities to gather and disseminate adequate information to plug the suggested information gaps. In all, concerns and misconceptions regarding safety of HPV vaccination were reported to have initially undermined uptake of the vaccination, which is in agreement with numerous studies that report concern about safety of HPV vaccination being a disincentive for vaccination against HPV [2, 3, 9, 20, 22]. These findings suggest that efforts to promote vaccination against HPV should clearly articulate the non-serious nature of the vaccination’s known adverse effects, their remedies, and brevity. They should also include deliberate strategies to neutralize the negative rumours and misinformation about the vaccination. This study also captured a concern by sections of the community that vaccination against HPV contradicts their religious and cultural values, which was said to have contributed minimally to opposition to the vaccination against HPV. This finding supports other studies which report that parents with strong religious or cultural views are least likely to support HPV vaccination [10, 14]. It suggests a need for religiously and culturally sensitive strategies to overcome these barriers.

In all, perceived benefits of HPV vaccination and cues to action seem to have greatly outweighed the potential barriers to the vaccination. The initial misconceptions and concerns about HPV vaccination were largely overcome through massive mobilization and sensitization of communities prior to the introduction of the HPV vaccination and during the vaccinations; hence the relatively high HPV vaccination coverage in Ibanda for all three doses that was estimated at 90.5 and 88.9% for the first and second years of vaccination, respectively [26]. Findings from the study however showed some lingering concerns and misconceptions about HPV vaccination of adolescent girls that potentially threaten sustained acceptability of HPV vaccination.

### Study limitations

There was a possibility of recall bias since data for this study was collected 1 year after vaccination of the P 6 girls. Selection of FGD participants with the help of teachers targeting girls and parents/guardians sharing certain characteristics may have biased the results since those girls and parents/guardians could have shared attitudes associated with shared characteristics. The effect of this was minimized by organizing FGDs in five different schools. This being a qualitative study, sampling was purposive and its findings cannot be generalized to the general population. The cause-effect relationship between the different perceptions about the HPV vaccine and their reported implications could not be assessed in a qualitative study.

## Conclusions

The perceived benefits and safety of HPV vaccination as well as understanding of the sexual transmission of HPV enhanced girls’ and parents’ acceptability of HPV vaccination. The Initial rumors, fears and concerns about HPV vaccination that reportedly discouraged some girls and parents, seemed to have waned with time giving way to more favourable perceptions regarding HPV vaccination although the study still found that a few concerns still lingered on and these have implications for HPV vaccination acceptability.

## Abbreviations

ACIP: Advisory Committee on Immunization Practices
CDP: Child Days Plus
CHS: College of Health Sciences
FGD: focus group discussion
HBV: hepatitis B virus
HIV: human immune deficiency virus
HN: hemagglutinin neuraminidase
HPV: human papillomavirus
KI: key informant
KII: key informant interview
LC: local council
NGAA: Next Generation of African Academicians
PATH: Program for Appropriate Technology for Health
P 5: primary five
P 6: primary six
RA: research assistant
STI: sexually transmitted infection
UNEPI: Uganda National Expanded Program for Immunization
